# Effect of Habitat Conditions and Plant Traits on Leaf Damage in the Carduoideae Subfamily

**DOI:** 10.1371/journal.pone.0064639

**Published:** 2013-05-22

**Authors:** Zuzana Münzbergová, Jiří Skuhrovec

**Affiliations:** 1 Department of Botany, Faculty of Science, Charles University, Praha, Czech Republic; 2 Institute of Botany, Academy of Sciences of the Czech Republic, Průhonice, Czech Republic; 3 Department of Plant Ecology and Weed Science, Crop Research Institute, Praha, Czech Republic; MESC; University of South Alabama, United States of America

## Abstract

Plant traits are the key factors that determine herbivore foraging selection. The traits serving as defense traits against herbivores represent a wide range of traits, such as chemical, physiological, morphological and life-history traits. While many studies considered plant defense traits at the within-species scale, much less is known from comparisons of a wide range of closely related species. The aim of this study was to identify factors responsible for the intensity of leaf damage in the Carduoideae subfamily of Asteraceae, which hosts many invasive species and thus is potential candidate plant species that could be controlled by biological control. Specifically, we wanted to see the relative importance of habitat characteristics, plant size and plants traits in determining the degree of folivory. The study identified several defense traits able to explain differences in herbivory between species after accounting for differences in the habitats in which the species occur and the plant size. Specifically, the most important traits were traits related to the quality of the leaf tissue expressed as the content of phosphorus, water and specific leaf area, which suggests that the leaf quality had a more important effect on the degree of herbivory than the presence of specific defense mechanisms such as spines and hair. Leaf quality is thus a candidate factor that drives herbivore choice when selecting which plant to feed on and should be considered when assessing the danger that a herbivore will switch hosts when introduced to a new range.

## Introduction

Plant traits are key factors that determine herbivore selection of forage, and the herbivores can play a key role in the evolution of these plant traits [1], [2], [3], [4]. The traits serving as defense traits against herbivores include a wide range of traits, such as chemical, physiological, morphological and life-history traits [5], [6], [7], [8], [9], [10]. In a recent review, [10] tested the importance of these traits for plant-herbivore interactions and concluded that morphological and physical traits are often more important for plant-herbivore interaction than chemical traits. Their study was based on a review of 66 published studies where correlations between plant traits and herbivore attacks were tested at the microevolutionary (within species) scale.

In contrast to the high number of studies on this topic at the microevolutionary scale, [10] identified only 6 studies that would allow a similar comparison at the macroevolutionary (between-species) scale. Out of these studies, only one, [11], compared the intensity of herbivory in a larger number of closely related species. They studied 32 species of the Onagraceae family and tested the relationship between sexuality in the species and susceptibility to arthropod herbivores and detected a strong relationship between these factors. Data such as these may provide valuable background data for predictions of possible associations between plants and herbivores for the purpose of biological control by indicating which might be the key traits that determine the susceptibility of the plant species to various herbivores. We are, however, not aware of any study that would attempt to do this.

In this study, we consider the association between plant traits and the intensity of herbivore attacks within the Carduoideae subfamily of Asteraceae. This species group is of interest because many strongly invasive species (e.g., *Cirsium arvense*, *Cirsium vulgare*, *Centaurea maculosa, Carduus nutans*) originate from this subfamily [12], [13], [14], [15]. In addition, the biological control of these species is being used mainly in the United States [16], and the escape of biological control agents to other plant species from this group has been previously reported [17]. All of these factors suggest that the Carduoideae subfamily is an ideal candidate system to identify the traits responsible for the association between the plant species traits and the intensity of herbivore attacks.

The association between plants and herbivores is not dependent only on the traits of the plant. The habitats, including the composition of the surrounding vegetation, in which the species occur may also act as important determinants of the interactions of plants with their herbivores [18], [19], [20], [21], [22]. As a result, the intensity of herbivore damage will depend on the interaction between the plant traits and the habitat conditions. We are, however, not aware of any study that explores the relative importance of plant traits and habitat conditions for plant-herbivore interactions within larger species groups.

The aim of this study was to identify factors responsible for the intensity of leaf damage in the Carduoideae subfamily. Specifically, we wanted to observe the relative importance of habitat characteristics and the plants traits in determining the degree of folivory and answer the following questions. 1) What is the relationship between the intensity of plant damage in the field and habitat conditions in which the plants are growing? 2) What is the effect of plant size on the intensity of plant damage after accounting for the effect of habitat conditions? 3) What is the effect of species traits after accounting for the possible confounding effects of habitat conditions and plant size?

## Methods

### Study species and study localities

For the purpose of the study, we selected all of the species of Carduoideae occurring in the Czech Republic, Europe, that are sufficiently common to be studied (excluding species with only a few populations; unknown distributions, e.g., due to taxonomical difficulties; and species that are protected by the law), which resulted in a set of 32 species (Table 1). For each species, we selected 3 populations within the Czech Republic that were at least 20 km apart from each other, resulting in 96 studied populations in total. No specific permits were required for the fieldwork described.

**Table 1:** List of species used in the study.

*Arctium lappa*
*Arctium minus*
*Arctium nemorosum*
*Arctium tomentosum*
*Carduus acanthoides*
*Carduus crispus*
*Carduus nutans*
*Carduus personata*
*Carlina acaulis*
*Carlina vulgaris*
*Centaurea cyanus*
*Centaurea jacea*
*Centaurea macroptilon* subsp. *oxylepis*
*Centaurea maculosa*
*Centaurea phrygia* 2×
*Centaurea phrygia* 4×
*Centaurea phrygia* subsp. *pseudophrygia*
*Centaurea scabiosa*
*Centaurea triumfetti*
*Cirsium acaule*
*Cirsium arvense*
*Cirsium canum*
*Cirsium eriophorum*
*Cirsium helenioides*
*Cirsium oleraceum*
*Cirsium palustre*
*Cirsium pannonicum*
*Cirsium rivulare*
*Cirsium vulgare*
*Echinops sphaerocephalus*
*Onopordum acanthium*
*Serratula tinctoria*

The nomenclature of the species is unified according to Flora Europaea (web 1), and 2× and 4× indicate the use of diploid and tetraploid cytotypes of the given species – each cytotype is treated as a separate species.

### Folivores at the localities

The populations studied host a wide range of folivores. True folivory can be caused by invertebrates from several groups. Specialist (monophagous or oligophagous) folivores known for plants from the subfamily Carduoideae in the Czech Republic are mainly caterpillars (e.g., *Anania perlucidalis* (Hübner, 1809) (Lepidoptera: Crambidae), *Jordanita subsolana* (Staudinger, 1862) (Lepidoptera: Zygaenidae), *Pyroderces argyrogrammos* (Zeller, 1847) (Lepidoptera: Cosmopterigidae)) and also the larvae of beetles (e.g., *Cassida rubiginosa* Müller,1776 (Coleoptera: Chrysomelidae) [23], [24]. True folivory is caused also by generalist invertebrates, such as some caterpillars (e.g., *Autographa gamma* (Linnaeus, 1758) (Lepidoptera: Noctuidae)), grasshoppers, or some slugs (e.g., *Arion lusitanicus* Mabille, 1868).

Leaves are also damaged by sap insect (Hemiptera: Aphididae (e.g., *Uroleucon aeneus* (Hille RisLambers, 1939)); Hemiptera: Cicadellidae (e.g., *Eupteryx notata* (Curtis, 1837)) or leaf miners (e.g., *Phytomyza autumnalis* Hering, 1957 (Diptera: Agromyzidae), *Agonopterix arenella* (Denis & Schiffermüller, 1775) (Lepidoptera: Depressariidae), *Coleophora therinella* Tengstrom, 1848 (Lepidoptera: Coleophoridae), *Lobesia absciana* (Doubleday, 1849) and *Pelochrista modicana* (Zeller, 1847) (both Lepidoptera: Tortricidae) [23], [24].

### Habitat conditions

We recorded the presence of all of the common plant species within each population (within an area of approximately 400 m^2^) and used the information to describe the habitat conditions at the sites. We did not record the species present only as a few individuals in a vegetative stage. We used the information on the presence of the plant species to calculate the mean Ellenberg indicator values for each locality and used this information to describe the habitat conditions at the localities. The Ellenberg indicator values for each species were obtained from [25]. These values express the relationships of plant species to five environmental variables (light, temperature, moisture, soil reaction and nutrients), which are measured on a 9-degree ordinal scale (except moisture with 12-degree scale). The Ellenberg indicator values have previously been used in a wide range of studies and were shown to predict habitat conditions in a wide range of sites well e.g., [26], [27], [28], [29]. All of the species of Carduoideae were excluded from the calculation of the mean Ellenberg indicator values to ensure that the information on the habitat conditions was independent of the presence of the species from the Carduoideae subfamily studied. By calculating mean Ellenberg indicator value for each studied species, we in fact calculated the Ellenberg indicator value for the given studied species. While we could simply use the indicator value for the given species as provided by [25], we preferred to calculate this value based on species composition at the sites to capture conditions at the studied localities and not at places where the species usually occurs. In addition, the Ellenberg indicator values are not available for all of the species studied. Some additional species are considered not to have preferences for some environmental factors, and the values are thus not defined. The Ellenberg indicator value for each species and the calculated Ellenberg indicator value based on the species composition of the sites were significantly correlated in all cases except for light (r value ranging between 0.19 and 0.75).

### Plant traits

We collected 1 healthy undamaged leaf from the bottom part of the plant from 20 flowering plants per locality. We estimated the fresh and dry weight of these leaves and their area. We used this information to calculate the specific leaf area (SLA) of the leaves as the area divided by the dry weight (mm^2^/g). We also calculated the leaf water content by dividing the fresh leaf weight by the dry leaf weight.

To describe the degree of leaf dissection, we divided the leaf area by the area of the smallest possible smooth shape that could be drawn around the leaf.

A mixture of the leaves was used to estimate the contents of nitrogen, carbon and phosphorus in the leaf biomass. The chemical analyses were performed in the Analytical laboratory of the Institute of Botany, Academy of Sciences of the Czech Republic. The contents of nitrogen and carbon were analyzed following [30]. The content of phosphorus was analyzed spectrophotometrically at a wavelength of 630 nm (Unicam UV4-100, Cambridge, UK; [31] after digestion in HNO_3_ and H_2_O_2_. We also expressed the ratio between the carbon and the nitrogen content as another trait, the C/N ratio.

To estimate the leaf toughness, we used 10 leaves from each population and measured the toughness of each leaf 3 times while avoiding the veins. The measurements were averaged. The leaf toughness correlates with fiber and lignin and can be estimated by measuring the force needed to penetrate a leaf sample or the force needed to tear apart a leaf sample [32], [33]. We used a simplified penetrometer described in [33] to do the measurements. Specifically, we used a wire connected to a metal plate, a battery and a light bulb. The metal plate was placed on a balance with a precision of 0.001 g, and the leaf was placed on the metal plate. We measured the weight needed to punch through the leaf as indicated by lighting up the light bulb.

To estimate the spinosity of the leaves we counted the number of spines on the leaf margin and expressed it as the number of the spines per 1 cm of the leaf margin. We also measured the length and area of the spines. To measure the spine toughness, we classified the spines into 4 categories from soft to very tough.

To estimate the leaf hairiness, we used 1 leaf from 10 different individuals from each population and counted the number of hair on a section of the main vein. The hairiness was expressed as the number of hairs per 1 mm of the vein. We also measured length of 3 hairs per vein and averaged the numbers. To estimate the hairiness on the leaf blade, we classified the leaf according to hairiness in categories ranging from not at all hairy (0) to (4) completely cover by hair. This work was performed separately for upper and bottom part of the leaf. We also summed these two values to obtain the overall hairiness of the leaf blade. In addition we estimated the length of the hairs using a scale from 1 to 4.

While it would be ideal to collect the data on plant traits for all of the individual plants studied, it was not feasible due to the high complexity of collecting the data on the different plant traits. In addition, the data on plant traits were in a few cases even collected in different localities than the data on plant damage (these populations were always within 5 km from the target population but could be smaller and not suitable for collecting the data on herbivory). This circumstance occurred in cases when we did not collect the data on the traits on the same occasion as the sampling data on herbivore damage, and it was not possible to collect the data on the next visit (e.g., because the population was mown or the plants were already withered). We thus decided to concentrate on obtaining good estimates of the mean trait values per species rather than on more detailed data per population or per individual.

### Plant damage

To estimate the degree of leaf damage per plant, we randomly selected 50 flowering plants per locality along one or several transects to cover the whole locality. The length of the transects depended on the size and shape of the populations. We never selected plants less than 1 m from each other (the plants were separated by 5 m in the case of large populations). In some cases, multiple transects were used (at least 5 m from each other) to obtain a sufficient number of individuals to be studied. For each species, this information was collected at the time of peak flowering. For each plant, we measured its height and the number of flower heads and counted the number of undamaged leaves, the number of leaves with damage less than 10%, the number of leaves with damage less that 50% and the number of leaves with damage over 50%. We used this information to estimate the proportion of damaged leaves, the proportion of leaves with more than 50% damage and the overall degree of leaf damage. The overall degree of leaf damage was estimated as the mean damage per leaf per plant. The leaves with no damage were given a weight of 0, leaves with up to 10% were given a weight of 0.05, leaves up to 50% were given a weight of 0.3, and leaves over 50% were given a weight of 0.75. The sum of the values was divided by the total number of leaves per plant. A value equal to 0 indicated that no leaves were damaged, and a value of 0.75 indicated all leaves had more than 50% damage. Because of a strong correlation between the proportion of leaves with more than 50% damage and the overall degree of leaf damage (r = 0.96), we eventually used only the proportion of damaged leaves and the overall degree of leaf damage in the tests.

### Data analyses

The unit of observation in our analyses was species, and we thus analyzed all of the data at the species level by using species means. We use species means rather than more detailed data because different data were collected at different levels. Specifically, data on plant damage and plant size were collected at the level of individuals, but data on the plant traits and the habitat conditions were collected at the population level. In our study, we however, aim to view all the characteristics as characteristics of single species. In all cases, we used generalized linear models with a quasi-binomial distribution to analyze the data. The dependent variables were the proportion of damaged leaves and the overall degree of leaf damage.

The statistical models explaining leaf damage in the field were built using a multi step procedure. First, we used a principal component analysis to analyze the relationship between the variables describing habitat conditions. The habitat conditions were expressed as the Ellenberg indicator values for the light availability, the moisture availability, the nutrient availability, the pH and the temperature. The PCA indicated that the first gradient in the data increases from localities with high pH, temperature and light to localities with high moisture. The second major gradient was represented by the gradient of nutrient availability. Thus, we selected only the nutrient availability and the moisture as the two variables to be tested as habitat conditions. We tested the effect of these habitat conditions on the leaf damage. When they were significant, they were included in the subsequent models.

Afterwards, we performed a principal component analysis to analyze the relationship between the variables describing plant size, i.e., the number of flower heads per plant, the plant height and the number of leaves. All of these three variables were highly correlated, with the number of leaves being the most correlated with the first PCA axis. We tested the effect of the number of leaves on plant damage using a model that also included the habitat characteristics selected as significant in a previous step. When it was significant, it was included in the subsequent models.

In the third step, we performed a principal component analysis using the data on the plant traits. Afterwards, we used two types of tests. In one type, we preselected 10 traits of the original 18 traits based on their correlations. When selecting among the traits, we always preferred more composite traits (e.g., overall leaf hairiness) over simpler traits (e.g., hairiness on the upper part of the leaf). The final traits used in the analyses included the specific leaf area, the water content in fresh leaves, the leaf toughness, the phosphorus content in the leaves, the C/N ratio in the leaves, the leaf dissection, the spine length, the number of spines per leaf margin area, the spine toughness and the overall leaf hairiness. We used a step wise both directional procedure to select the optimal model but kept the previously included information on habitat conditions and plant size in the baseline model. Using this procedure, we considered the habitat conditions as the primary drivers of leaf damage followed by plant size. The plant traits are considered only after considering the habitat conditions and plant size.

The effect of the traits was also tested using an alternative approach. Specifically, we replaced the single species traits by the species positions in the first four PCA axes based on the plant traits and used these positions in the subsequent tests instead of the traits themselves.

All of the univariate analyses were performed using R 2.14.1 [34], and all the multivariate analyses (PCA) were performed using Canoco 5.0 [35].

## Results

**Figure 1:**
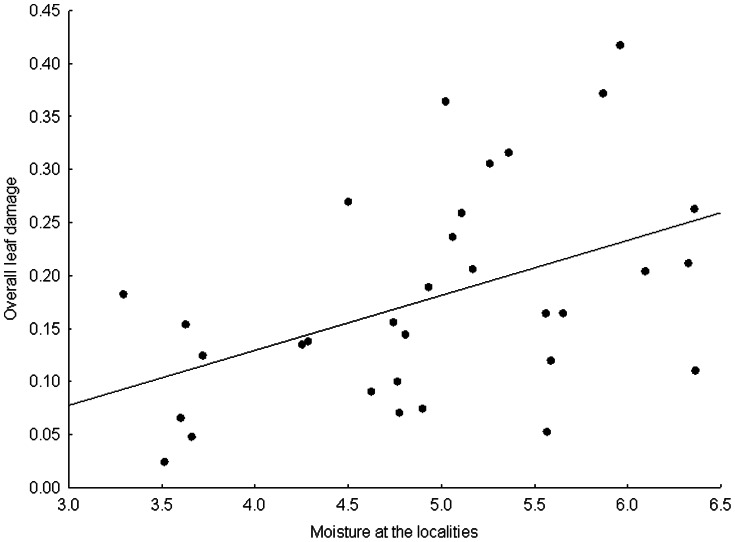
Effect of the moisture at the habitat on the overall leaf damage.

The proportion of damaged leaves and the overall leaf damage significantly increased with increasing moisture of the habitats occupied by the species studied (Figure 1). In addition, the proportion of damaged leaves also significantly increased with the nutrient availability at the habitats (Table 2A).

**Table 2:** Effect of the habitat conditions (moisture and nutrient availability), the plant size (no. of leaves) and the species traits on the proportion of damaged leaves.

	Proportion of damaged leaves				Overall leaf damage			
	F	p	R^2^	sign	F	p	R^2^	sign
Habitat moisture	**13.7**	**<0.001**	**0.12**	+	**11.8**	**<0.001**	**0.21**	+
Habitat nutrient	**20.1**	**<0.001**	**0.17**	+				
No. of leaves	**20**	**<0.001**	**0.17**	−				
A)								
Specific leaf area					**8.16**	**0.01**	**0.14**	+
Leaf water content	*5.44*	*0.03*	*0.05*	+	**6.5**	**0.02**	**0.11**	+
Leaf phosphorus	**17**	**<0.001**	**0.14**	+	*4.03*	*0.05*	*0.07*	+
Spine length	3.82	0.06	0.03					
Spine toughness	**11.2**	**<0.001**	**0.09**	+				
Leaf dissection	2.84	0.11	0.02					
B)								
PCA axis 1	0.16	0.69	<0.001		0.85	0.36	0.02	
PCA axis 2	0.45	0.51	0.01		0.42	0.52	0.01	
PCA axis 3	**7.19**	**0.01**	**0.10**	−	0.70	0.41	0.02	
PCA axis 4	**9.03**	**0.01**	**0.13**	**+**	**7.32**	**0.01**	**0.16**	+

**Figure 2:**
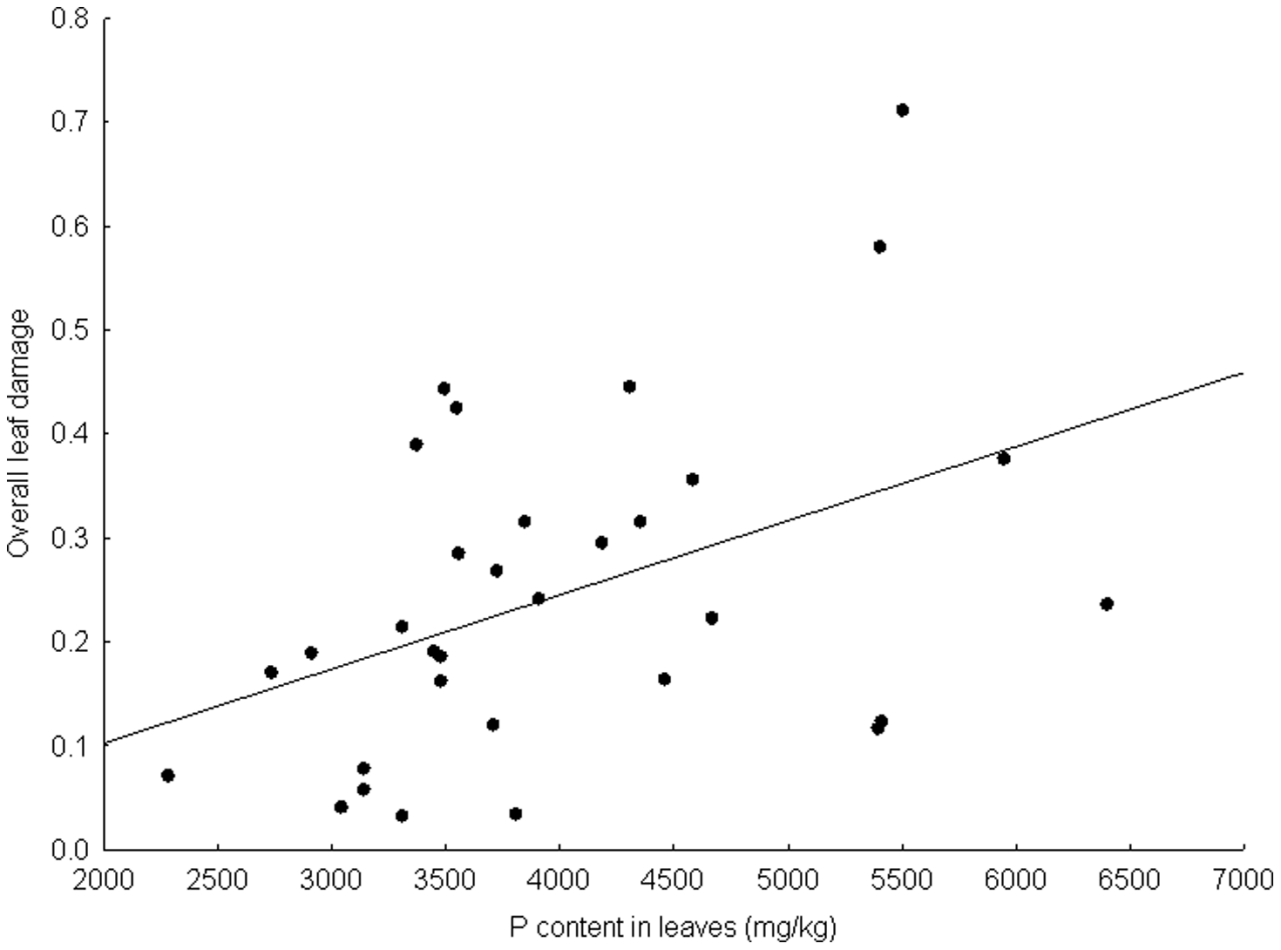
Effect of the content of phosphorus in plant leaves on the overall leaf damage.

Two different tests using the plant traits were performed. A) The traits were selected using a step-wise both directional procedure, and only variables included in the final model are shown. B) Traits were summarized using principal component analysis (Figure 2), and the positions of species on the first, second, third and fourth axes were used as independent variables.

After considering the differences in habitat conditions, the proportion of damaged leaves was significantly lower in species with a higher number of leaves while the overall leaf damage was independent of the plant size (Table 2A).

**Figure 3:**
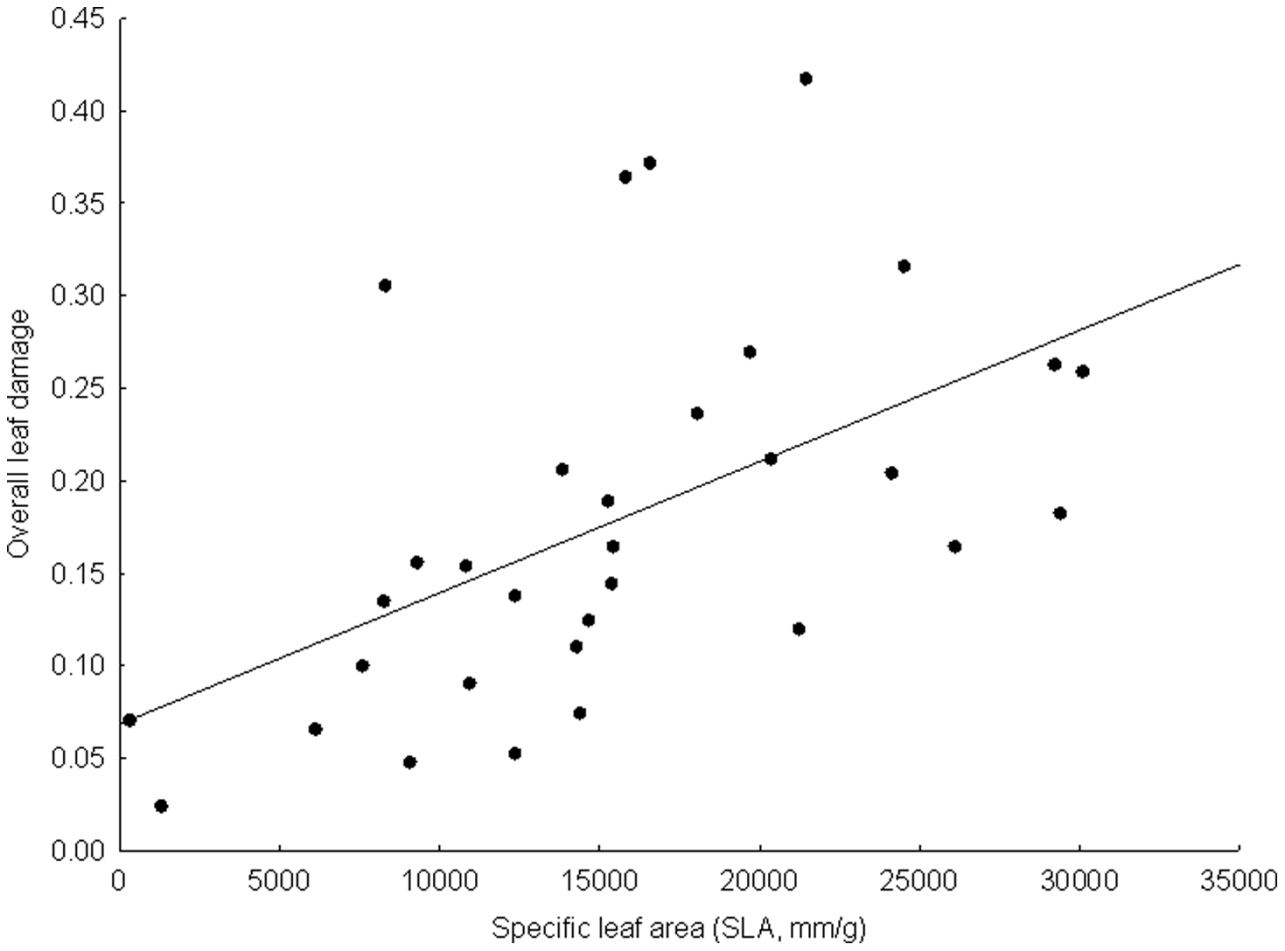
Effect of the specific leaf area on the overall leaf damage.

After accounting for the habitat conditions and the plant size, both the proportion of damaged leaves and the overall leaf damage increased with increasing content of water in fresh leaves and with increasing content of phosphorus (Figure 2). The overall leaf damage also increased with increasing specific leaf area (Figure 3). In contrast, the proportion of damaged leaves increased with increasing spine toughness (Table 2A).

Overall, the habitat conditions explained 29% and 21% of the proportion of damaged leaves and overall leaf damage, respectively, and the plant size and the plant traits explained 17% and 0% and 33% and 32% of proportion of damaged leaves and overall leaf damage, respectively (Table 2A).

**Figure 4:**
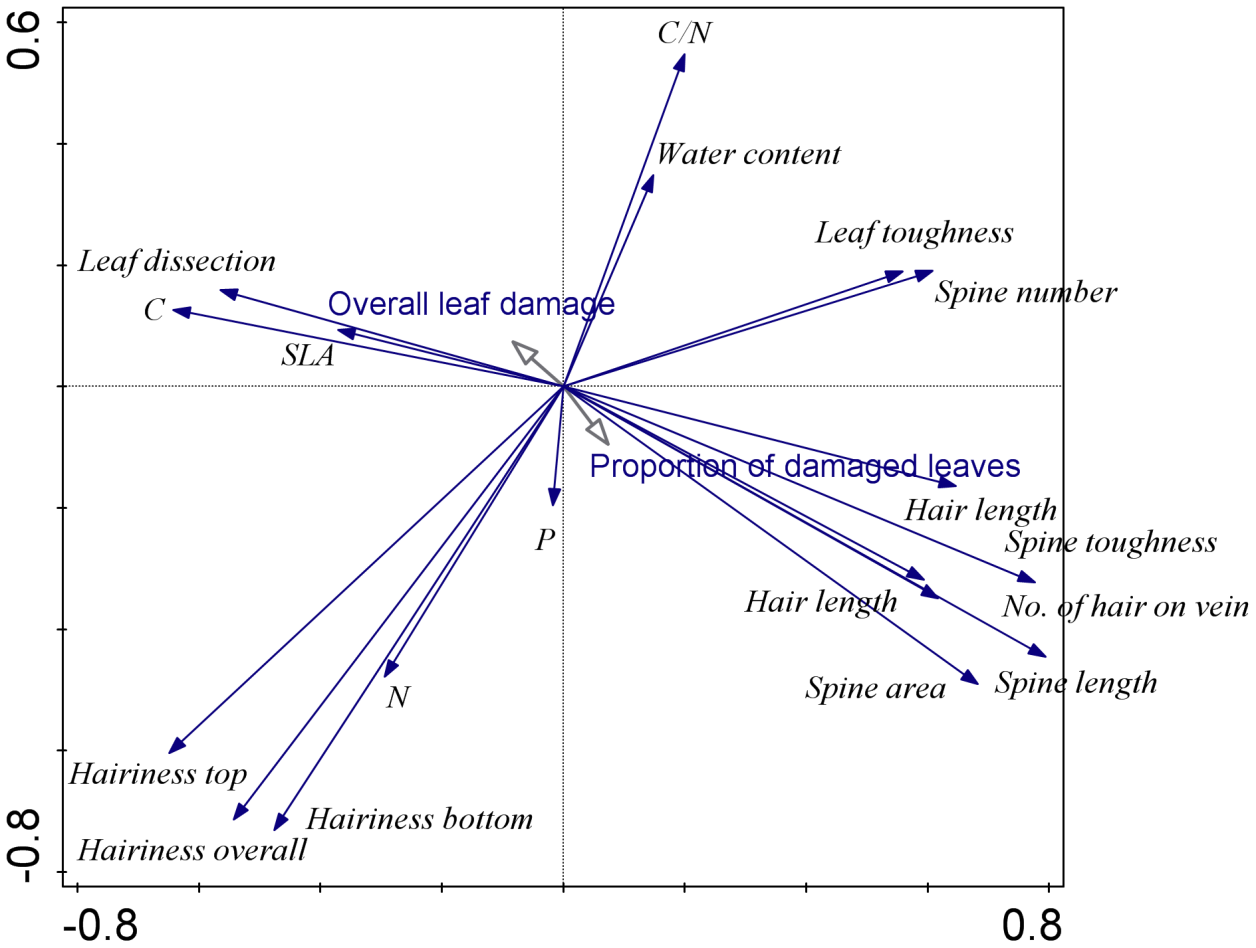
Results of the principle component analysis with species traits as the dependent variables and the overall leaf damage and the proportion of damaged leaves as supplementary variables. The first (horizontal) and the second (vertical) ordination axes explain 30% and 17.9% of the total variation, respectively.

**Figure 5:**
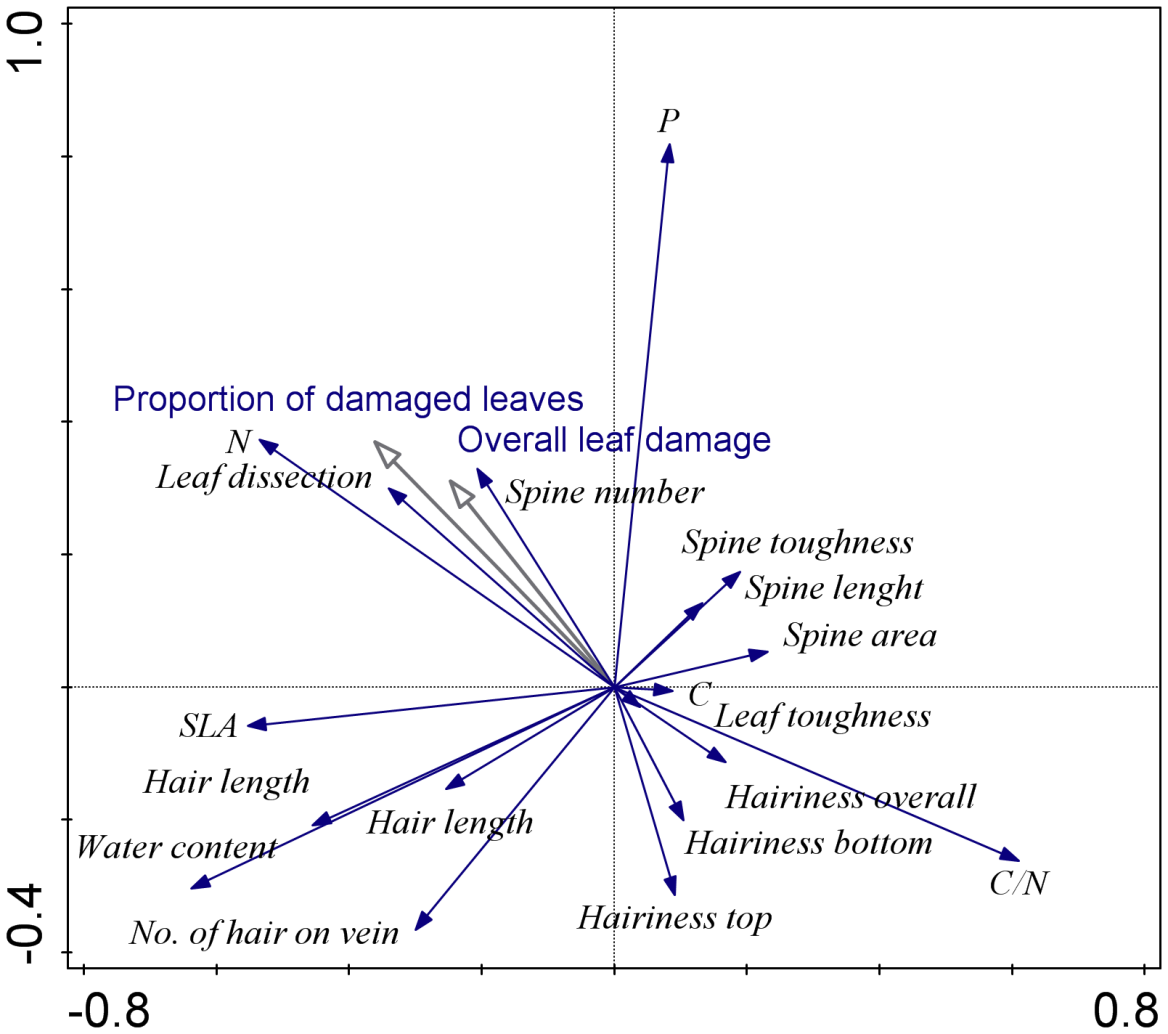
Results of the principle component analysis with species traits as the dependent variables and the overall leaf damage and the proportion of damaged leaves as supplementary variables. The third (horizontal) and the fourth (vertical) ordination axes explain 11.6% and 8.3% of the total variation, respectively.

The major gradient in the trait data based on principal component analysis ranged from plants with high leaf dissection, high carbon content and high hairiness to leaves with high spine toughness and high spine number. This gradient explained 30% of the total variation in the trait data. The second gradient ranged from leaves with high C/N ratio and high water content to leaves with high hairiness. This gradient explained 17.9% of the total variation in the data (Figure 4). The third gradient ranged from leaves with high nitrogen content and high specific leaf area to leaves with high C/N ratio. This gradient explained 11.6% of the total variation in the data. The fourth gradient was the gradient of the phosphorus content in the leaves, and it explained 8.3% of the total variation in the data (Figure 5).

When including the positions of the species on the PCA axes in the models, including the significant effects of the habitat conditions and the plant size, the proportion of damaged leaves was significantly affected by the species position on the third and the fourth axes. The overall leaf damage was significantly affected only by the species position on the fourth axis (Table 2B). The plant traits represented by the position of the species on the PCA axes explained 23% and 16% of the proportion of damaged leaves and the overall leaf damage, respectively.

## Discussion

The proportion of damaged leaves was significantly explained by all three groups of variables, i.e., the habitat conditions, the plant size and the plant traits. The habitat conditions and the plant traits explained very similar proportions of the total variation while the variation explained by the plant size was lower. In contrast, the overall leaf damage was independent of the plant size, and the plant traits explained more of the variation than the habitat conditions. The predictive power of the plant traits summarized using PCA analysis was lower than the predictive power of the single traits.

The significant relationship between the leaf damage and the habitat conditions is in agreement with many previous studies that indicate that plants tend to suffer higher herbivore damage in wetter, nutrient richer and more shaded habitats e.g., [36], [37], [38], [21], i.e., in habitats in which they often tend to grow more vigorously. Such an effect was previously described as the Vigor Hypothesis, which states that the plants from the most vigorous populations, the most vigorous plants within a population and/or the most vigorously growing parts within a plant should suffer higher herbivore damage [39]. Alternatively, the habitat conditions may directly affect populations of the herbivores by providing them better conditions for growth and/or reproduction, as was previously demonstrated for a wide range of herbivores e.g., [40], [41], [42]. The strong relationship between herbivore damage and habitat conditions supports the importance of considering the habitat conditions in studies on the effect of herbivores on plant performance.

After considering the habitat conditions in which the plants occur, the proportion of damaged leaves decreased with an increasing number of leaves while the overall leaf damage was unaffected by the plant size. Previous studies relating the plant size and herbivore damage found both higher herbivore damage on the larger plants and the opposite pattern e.g., [43], [44], [45], [21]. The pattern found in our study may be related to the individual plant growth rate because plants growing faster and thus producing new leaves faster are more likely to have some leaves undamaged by herbivores at any moment in time. This explanation also fits with the fact that the overall plant damage is independent of the plant size, suggesting that even these vigorously growing plants with many new undamaged leaves may be otherwise strongly damaged by the herbivores.

Species with higher water and phosphorus contents in the leaves had a higher proportion of damaged leaves and higher overall leaf damage. The content of phosphorus is one of the key limiting nutrients for a wide range of herbivores [46]. Increased herbivory with increasing phosphorus concentration in the leaves was reported in studies on the effects of fertilization by phosphorus in tropical rain forests [47], [48]. In addition, the water content in leaves has already been previously reported as a possibly important factor for increasing the attractivity of foliage to herbivores [46].

Surprisingly, the proportion of damaged leaves also increased with spine toughness. The opposite pattern, i.e., lower leaf herbivory with increased spine toughness, would be expected in the case of herbivory by large herbivores e.g., [49], [50], [51], [52], [53]. The main herbivores in our study system were, however, invertebrates, as described in the methods. Their feeding is clearly independent of spines, and their preference to leaves with higher spine toughness may be a result of their preference to leaves of higher quality, in which the selection pressure on protection by spines is higher [53].

The overall leaf damage was also higher in plants with a higher specific leaf area. In previous studies, the specific leaf area was shown to be a good predictor of the growth rate of plants e.g., [54], [55], and plants with higher growth rate were predicted to suffer from higher herbivory [39]. In addition, leaves with higher specific leaf area are expected to have a lower tissue density, thinner leaf lamina and weaker veins [56] and be less tough [57], which makes them more palatable to herbivores. Indeed, several studies found a positive relationship between the specific leaf area and herbivory e.g., [58], [59], but see [57], [60], [61] for the lack of a relationship).

In contrast to the traits discussed above, there were several traits that we expected to be associated with herbivore damage, but no such relationship was detected in the step-wise analysis. These traits include the leaf toughness, the C/N ratio in leaves, the leaf dissection, the spine length, the number of spines per leaf margin area and the overall leaf hairiness. The lack of the effect of these traits can be partly attributed to the fact that they are correlated with the traits that were selected as having an effect, which could be the case for leaf dissection and the number of spines, both of which were significantly positively correlated with spine toughness. The overall leaf hairiness was also negatively correlated with the water content in the leaves. In addition, several of these traits may be identified as not significant due to the low power of our tests (high probability of type II errors due to the relatively small number of data points, i.e., species). Their possible effects can, however, be seen from their correlation with leaf damage in the PCA analysis. Specifically, leaf damage seems to be positively correlated with leaf dissection and spine number and negatively with C/N ratio and leaf hairiness.

Because a high nitrogen content in biomass is important to the herbivore diet [62], a high C/N ratio can have a negative effects on herbivore performance [63]. The negative effects of a higher C/N ratio and the positive effects of a higher nitrogen content in leaves were previously reported in several studies e.g., [59], [64]. The lack of a relationship in our study may be attributed to the fact that the phosphorus concentration in leaves was most likely more limiting than nitrogen because both of these nutrients may limit insect performance and the limitation by one or the other depends on the environment [62]. Alternatively, the absence of an effect of the nitrogen content may be due to weak power of the test, as mentioned above.

Another important trait to decrease plant herbivory was expected to be leaf toughness. Lower herbivory with increasing leaf toughness was previously reported in many studies e.g.,[65], [66], [67], [68], but other studies report the absence of any relationship in agreement with our study e.g., [69]. This discrepancy is likely to depend on the range of tested species, with a relatively narrow range of leaf toughness values among our species coming from the same subfamily. No relationship between the leaf damage and the leaf toughness was apparent from the principal component analysis, and the absence of this relationship is thus likely not due to type II errors.

In addition, the length of the spines was expected to be related to herbivory based on previous studies e.g., [53], but no such pattern was in fact detected in our study. This discrepancy could be explained by prevailing herbivory by invertebrates that can easily feed among the spines. No relationship between the leaf damage and the spine length was apparent from the principal component analysis, and the absence of this relationship is thus likely not due to a type II error.

An important motivation for this study was to understand the potential drivers of herbivore choice in an important plant group that hosts many invasive species. Recently, biological control has been used for the control of several of these species, mainly in the United States [16] and the escape of biological control agents to other species from this group has been previously reported [17]. The results of this study suggest that the leaf quality may be one of the key plant traits that drives herbivore choice when a new herbivore is moved into a new range. Future studies working with species from the Carduoideae subfamily should thus concentrate on assessing leaf quality in species native to North America. This knowledge will aid our ability to predict the risk of escape of biological control agents to these native species.

## Conclusions

The study identified several defense traits able to explain differences in herbivory between species in the Carduoideae subfamily of Asteraceae after accounting for differences in habitats in which the species occur and for plant size. Specifically, the most important traits were traits related to the quality of the leaf tissue, which was expressed as the contents of phosphorus and water and the specific leaf area. This result suggests that leaf quality is a more important mechanism that affects the degree of herbivory than the presence of specific defense mechanisms, such as spines. Leaf quality is thus a candidate factor that drives herbivore choice when selecting which plant to feed on and should be used when judging the danger that a biocontrol agent will escape to alternative hosts.
